# Behavioral skills training for teaching safety skills to mental health service providers compared to training-as-usual: a pragmatic randomized control trial

**DOI:** 10.1186/s12913-024-10994-1

**Published:** 2024-05-17

**Authors:** Elizabeth Lin, Mais Malhas, Emmanuel Bratsalis, Kendra Thomson, Fabienne Hargreaves, Kayle Donner, Heba Baig, Rhonda Boateng, Rajlaxmi Swain, Mary Benisha Benadict, Louis Busch

**Affiliations:** 1 Department of Education, Centre for Addiction and Mental Health, Toronto, ON Canada; 2 Department of Applied Disability Studies, Brock University, St. Catharines, ON Canada

**Keywords:** Workplace violence, Violence prevention, Behavioural skills training, Performance and competency-based staff training

## Abstract

**Background:**

Violence in the healthcare workplace has been a global concern for over two decades, with a high prevalence of violence towards healthcare workers reported. Workplace violence has become a healthcare quality indicator and embedded in quality improvement initiatives of many healthcare organizations. The Centre for Addiction and Mental Health (CAMH), Canada’s largest mental health hospital, provides all clinical staff with mandated staff safety training for self-protection and team-control skills. These skills are to be used as a last resort when a patient is at imminent risk of harm to self or others. The purpose of this study is to compare the effectiveness of two training methods of this mandated staff safety training for workplace violence in a large psychiatric hospital setting.

**Methods:**

Using a pragmatic randomized control trial design, this study compares two approaches to teaching safety skills CAMH’s training-as-usual (TAU) using the 3D approach (description, demonstration and doing) and behavioural skills training (BST), from the field of applied behaviour analysis, using instruction, modeling, practice and feedback loop. Staff were assessed on three outcome measures (competency, mastery and confidence), across three time points: before training (baseline), immediately after training (post-training) and one month later (follow-up). This study was registered with the ISRCTN registry on 06/09/2023 (ISRCTN18133140).

**Results:**

With a sample size of 99 new staff, results indicate that BST was significantly better than TAU in improving observed performance of self-protection and team-control skills. Both methods were associated with improved skills and confidence. However, there was a decrease in skill performance levels at the one-month follow-up for both methods, with BST remaining higher than TAU scores across all three time points. The impact of training improved staff confidence in both training methods and remained high across all three time points.

**Conclusions:**

The study findings suggest that BST is more effective than TAU in improving safety skills among healthcare workers. However, the retention of skills over time remains a concern, and therefore a single training session without on-the-job-feedback or booster sessions based on objective assessments of skill may not be sufficient. Further research is needed to confirm and expand upon these findings in different settings.

**Supplementary Information:**

The online version contains supplementary material available at 10.1186/s12913-024-10994-1.

## Introduction

Violence in the healthcare workplace has been a global concern for over two decades. In 2002, a joint task force of the International Labour Office (ILO), World Health Organization, Public Services International, and the International Council of Nurses created an initiative to address this issue [1]. One result was the documentation of a high international prevalence of violence towards healthcare workers showing that as many as half or more experienced physical or psychological violence in the previous year [2, 3]. Since then, workplace violence has become a healthcare quality indicator and been embedded in the quality improvement initiatives of many healthcare organizations (for example, Health Quality Ontario [4]). Conceptually, it is also reflected in the expansion of the Triple Aim framework to the Quintuple Aim to include staff work-life experience [5].

Despite these efforts, the high prevalence of workplace violence in healthcare persists [6]. Two meta-analyses, representing 393,344 healthcare workers, found a 19.3% pooled prevalence of workplace violence in the past year among which 24.4% and 42.5% reported physical and psychological violence experiences, respectively [7, 8]. The literature also highlighted that workers in mental health settings were at particular risk [8, 9]. A systematic review of violence in U.S. psychiatric hospitals found between 25 to 85 percent of staff encountering physical aggression in the past year [10]. Partial explanations for this wide range include methodological, population, and setting differences. For example, Gerberich and colleagues [11] surveyed nearly 4,000 Minnesota nurses and found 13 percent reporting physical assault and 38 percent reporting verbal or other non-physical violence in the previous year. Further analyses showed that nurses on psychiatric or behavioral units were twice as likely as those on medical/surgical units to experience physical violence and nearly three times as likely to experience non-physical violence. Ridenour, et al., [12] in a hospital-record study of acute locked psychiatric wards in U.S. Veteran’s Hospitals found that 85 percent of nurses had experienced aggression in a 30-day period (85 percent verbal; 81 percent physical). And, in a prospective study of a Canadian psychiatric hospital, Cooper and Mendonca [13] found over 200 physical assaults on nurses within 27 months. While they do not indicate what percentage of nurses were assaulted, their results are consistent with a frequency of between 1 and 2 assaults per week.

Workplace violence has been associated with negative psychological, physical, emotional, financial, and social consequences which impact staff’s ability to provide care and function at work [14–16]. A 7-year, population-based, follow-up study in Denmark highlighted the long-term impact of physical and psychological health issues owing to physical workplace violence [17]. Two studies, one in Italy [18] and one in Pakistan [19], have linked workplace violence to demoralization and declining quality of healthcare delivery and job satisfaction among healthcare workers.

Building on these efforts, the ILO published a 2020 report recommending the need for national and organizational work environment policies and workplace training “…on the identified hazards and risks of violence and harassment and the associated prevention and protection measures….” ([20], p. 55). Consequently, many countries [21–23] have committed to creating a safe work environment. In Ontario, Canada, the government has provided guidelines for preventing workplace violence in healthcare [4, 24], and our institution, the Centre for Addiction and Mental Health, launched a major initiative in 2018 to address the physical and psychological safety of patients and staff [25]. A priority component of this initiative is mandatory training for all new clinical staff on trauma-informed crisis prevention, de-escalation skills, and, in particular, safe physical intervention skills [26, 27].

However, the effects of such training, especially for managing aggressive behaviour, are only partially understood. A 2015 systematic review on training for mental health staff [28] and a more recent Cochrane review on training for healthcare staff [29] reported remarkably similar findings. Both noted the inconsistent evidence (due to methodological issues, small numbers of studies, heterogenous results) which made definitive conclusions about the merits and efficacy of training difficult. The more consistent impacts found by Price and colleagues [28] were improved knowledge and staff confidence in their ability to manage aggression. There was some evidence of improved de-escalation skills including the ability to deal with physical aggression [30, 31] and verbal abuse [32]. However, these studies were limited because they used unvalidated scales or simulated, rather than real-world, scenarios. For outcomes such as assault rates, injuries, the incidence of aggressive events, and the use of physical restraints, the findings were mixed or difficult to generalize due to the inconsistent evidence.

Similarly, Geoffrion and colleagues [29] found some positive effect of skills-training on knowledge and attitudes, at least short-term, but noted that support for longer-term effects was less sure. The evidence for impacts on skills or the incidence of aggressive behaviour was even more uncertain. They also noted that the literature was limited because it focused largely on nurses. They concluded, “education combined with training may not have an effect on workplace aggression directed toward healthcare workers, even though education and training may increase personal knowledge and positive attitudes” ([29], p. 2). Among their recommendations were the need to evaluate training in higher-risk settings such as mental healthcare, include other healthcare professionals who also have direct patient contact in addition to nurses, and use more robust study designs. In addition, the literature evaluating training procedures focussed on self-reported rather than objective measures of performance.

Given the concerns with demonstrating effectiveness, the violence prevention literature has tended to focus on training modalities and immediate post-training assessment rather than on skill retention over time. In a systematic review of prevention interventions in the emergency room, Wirth et al. [21] found only five out of 15 included studies that noted any kind of evaluation in the period after training (generally two to nine months post-training) while Geoffrion, et al. [29] identified only two among the nine studies in their meta-analysis that had follow-up skills assessments. However, for both of these reviews, the studies doing follow-up evaluations focused on subjective, self-reported outcomes (empathy, confidence, self-reported knowledge) with no objective behavioral skills measures. Both Wirth et al. [21] and Leach et al. [33] cite studies noting a loss of effectiveness of prevention skills (between three to six months post-training), but specific percentages of retention were not provided.

The present study sought to address these gaps by comparing two approaches to teaching safety skills for managing aggressive patient/client behaviour. The setting was a large psychiatric teaching hospital; the sample was drawn from all new clinical staff attending their mandated on-boarding training; and we used a pragmatic randomized control trial design. In addition, we added a 1-month post-training assessment to evaluate skill retention. Our control intervention was the current training-as-usual (TAU) in which trainers “describe” and “demonstrate”, and trainees “do” by practicing the demonstrated skill but without objective checklist-guided performance assessment by the trainer. Our test intervention was behavioural skills training (BST) [34, 35] drawn from the field of applied behaviour analysis [36]. BST is a performance- and competency-based training model that uses an instructional, modeling, practice, and feedback loop to teach targeted skills to a predetermined performance level. Checklists guide the instructional sequence and the determination of whether or not the predetermined performance threshold has been reached. Considerable evidence indicates that BST can yield significant improvement in skills post-training, over time, and across different settings [37–39]. It has been used to train a wide range of participants, including behavior analysts, parents, and educators, to build safety-related skills and manage aggressive behavior [37, 40, 41].

## Methods

As previously described [42], our objective was to compare the effectiveness of TAU against BST. Our hypotheses, stated in null form, were that these methods would not differ significantly in:

1. Observer assessment of self-protection and team-control physical skills.
2. Self-assessed confidence in using those skills.

Study participants were recruited from all newly-hired clinical staff attending a mandatory two-week orientation. Staff were required to register beforehand for a half-day, in-person, physical safety skills session. They were randomized to a session at the time of registration, and the sessions were then randomized to TAU or BST. All randomization was performed by RB using GraphPad software [43].

The physical skills training was scheduled for a 3.5 h session on one day of the mandatory onboarding. At the end of the previous day, attendees were introduced to the study (including the fact that it was a randomized study) and asked for consent to email them a copy of the informed consent. On the morning of the physical skills training, a research team member met with attendees to answer questions and then meet privately with each individual to ascertain if they wished to participate and sign the informed consent. The trainers and session attendees were thus unaware of who was or was not in the study. Recruitment began January 2021, after ethics approval, and continued until September 2021 when the target of at least 40 study participants completing all assessments for each training condition was reached. The target sample size was chosen to allow 80-percent power to detect a medium to large effect size [44].

Both methods taught the same 11 target skills for safely responding to patients/clients that may exhibit harm to self or others (e.g., aggressive behaviour) during their hospital admission. These skills, defined by the hospital as mandatory for all newly hired staff, included six self-protection and five team-control (physical restraint) skills (see Appendix A). Each target skill had defined components and a specific sequence in which they were taught as outlined on performance checklists (see Appendix B for a checklist example).

The two methods differed in how these sequences were administered. For BST, the trainers used the performance checklists to guide the training sequence (instruction, modeling, rehearsal, and feedback) and to indicate when the trainee was ready to move on to the next skill [34] (see Appendix C for BST sequence). In BST, common practice is to define successful performance criteria a priori (e.g., up to three correct, consecutive executions at 100% [45]). However, because the physical skills training session in our study had to be completed within the scheduled 3.5 h, the criterion was lowered for practical reasons to one correct performance (defined as 80% of the components comprising that skill) with the added goal of aiming for up to 5 times in a row if time allowed before moving on to the next skill. In contrast, while TAU included elements of modeling, practice, and feedback, it did not systematically assess skill acquisition nor impose any specific level of success before proceeding to the next skill.

### Measures

There were three outcome measures, two observer-based assessments of skill acquisition (competence and mastery) and one self-reported confidence measure. Competence was defined as the percentage of components comprising an individual skill that were correctly executed (e.g., if a skill had 10 components and only six were executed properly, the competence score for that skill would be 60%). Mastery was the threshold defining when a competence score was felt to indicate successful achievement of a skill and to indicate some degree of the durability of the skill acquisition [46]. For our study, we expanded mastery to apply to the two categories of self-protection and team-control (rather than to each individual skill) using the average competence scores for the skills within each category. Mastery was pre-defined as 80-percent, a commonly used threshold [28, 47].

The outcome measures were assessed at three time points: immediately before training (baseline), immediately after training (post-training), and one month later (follow-up). The hospital provided limited descriptive information (professional role, department) for all registrants for administrative purposes but for confidentiality reasons did not provide personal information such as age or gender/sex. The research team elected not to collect personal information for two reasons. First, the primary study concern was to evaluate the main effect of training method rather than developing predictive models, and the expected result of the randomization process was that potential covariates would not be systematically biased in the two study groups. Second, we would not be able to use this information to compare participants with non-participants to identify biases in who consented to be in the study. We were able to compare them on department role and profession by subtracting the aggregated study-participant information from the aggregated hospital-provided information – the only form of the hospital-provided information available to the research team (see Table 1 below). In addition, since degree of patient contact was an important factor in the likelihood of needing to exercise safety skills, the research team also created an algorithm estimating which combinations of professional role and department were likely to have direct, less direct, or rare/low patient contact.

Participants were also asked at baseline and follow-up how many events they encountered in the previous month that required the use of these skills. This information was collected because of our interest in testing a post-hoc hypothesis that those with actual experience would score higher than those who did not.

All assessments were carried out following a standardized protocol. To ensure that registrants remained blinded to which colleagues were in the study, each registrant’s skill acquisition was assessed privately by a research team member at baseline and post-training using the performance checklists. Only assessments for those consenting to participate were videotaped. Study participants were then asked to return one month later for a follow-up assessment which was also videotaped. For the purposes of post-hoc analyses, participants completing all three assessments were defined as ‘completers’ while those completing baseline and post-training assessments but not the one-month follow-up were ‘non-completers.’

The same performance checklists used by the BST trainers were then used by trained observers blinded to the participant’s training method to assess the videotapes. As described previously [42], interobserver agreement (IOA) was routinely evaluated throughout the study with the final value being 96% across the 33% of the performance assessment videos scored for the IOA calculation.

Skill acquisition outcomes were calculated using the checklist-based observer assessments of the videotapes. The percentage of correctly executed components for each target skill was established. Then, these percentages were averaged across the six self-protection target skills and across the five team-control target skills to create competence scores. Finally, the predefined threshold of 80% was applied to the competence scores to determine which participants met the mastery threshold [47, 48].

Self-reported confidence was assessed on a 10-point Likert scale (‘not at all’ to ‘extremely’ confident) using a version of our institution’s standard assessment questions adapted for this study (See Appendix D).

### Statistical analysis

R software was used to generate descriptive statistics (frequencies, percentages) and test our hypotheses [49]. Generalized linear mixed models (GLMM) were used to test nested main and interaction effects using likelihood-ratio chi-square statistics for the post-training and follow-up results as there were no baseline differences. GLMM was also used to evaluate BST-TAU differences at the three study time points [50, 51]. For the BST-TAU comparisons, we used Cohen’s *d* as a guide for evaluating the practical significance of the differences for the continuous measures (competence, confidence). We used Cohen’s suggested thresholds [52] of 0.2, 0.5, and 0.8 for small, medium, and large effect sizes conservatively by applying them to both the point estimates and 95% confidence intervals. Thus, for example, a Cohen’s *d* where the confidence interval went below 0.2 would be interpreted as non-meaningful. For the categorical measure of mastery, we used BST-TAU risk ratios. Confidence intervals for all effect size measures were obtained using bootstrapping. Independent-samples *t*-tests were used for the post-hoc analyses and, along with chi-square tests, to compare the completers and non-completers.

## Results

One hundred ninety-nine staff consented to participate in the study out of a total of 360 session attendees (55%). Of these, 108 (54%) had been randomly assigned to a BST session and 91 (46%) to a TAU session. Half (*n* = 99) completed assessments at all three time points (44% TAU; 55% BST). These 99 (hereafter ‘study completers’) constituted 28 percent of all session attendees.

Among the non-completers, 53 had been assigned to BST and 47 to TAU. Eight were classified as incomplete because of technical software issues when video-recording one of their assessments and one (the first participant) because the IOA process prompted substantive changes to the assessment checklist. The primary reason for the remaining non-completers was missing the follow-up assessment (91 individuals: 50/53 BST, 41/47 TAU) largely due to difficulties scheduling a non-mandatory event during the pandemic (e.g., units restricting staff from leaving because of clinical staff shortages or patient outbreaks, staff illness).

Descriptive information for the expected degree of patient contact and for hospital department is shown in Table 1 for study participants (completers, non-completers), non-participants, and the total group of session attendees. No significant differences were found when comparing participants versus non-participants or study completers versus non-completers in terms of expected patient contact (*χ*^2^(2) = 0.36, n.s.; *χ*^2^(2) = 2.22, n.s.; respectively) or department type (*χ*^2^(3) = 4.40; (*χ*^2^(3) = 1.00, n.s.; respectively).

**Table 1:** Expected patient contact and department types for study participants, non-participants, and total session attendees

Characteristic	Study Participants (*n* = 199)		Non-Participants (*n* = 161)	Total Session Attendees (*n* = 360)
	**Completers** ***n* = 99**	**Non-Completers** ***n* = 100**		
**Expected Patient Contact: n (%)**				
Direct	93 (94)	97 (97)	155 (96)	345 (96)
Nurse	37 (37)	48 (48)	75 (47)	160 (44)
Security	6 (6)	5 (5)	5 (3)	16 (4)
Other	50 (51)	44 (44)	75 (47)	169 (47)
Less Direct	4 (4)	3 (3)	4 (3)	11 (3)
Rare/None	2 (2)	0 (0)	2 (1)	4 (1)
**Department Type: n (%)**				
Inpatient	57 (58)	64 (64)	82 (51)	203 (56)
Outpatient	9 (9)	9 (9)	23 (14)	41 (11)
Both	30 (30)	26 (26)	53 (33)	109 (30)
Hospital Admin	2 (2)	1 (1)	2 (1)	5 (1)

**Fig. 1:**
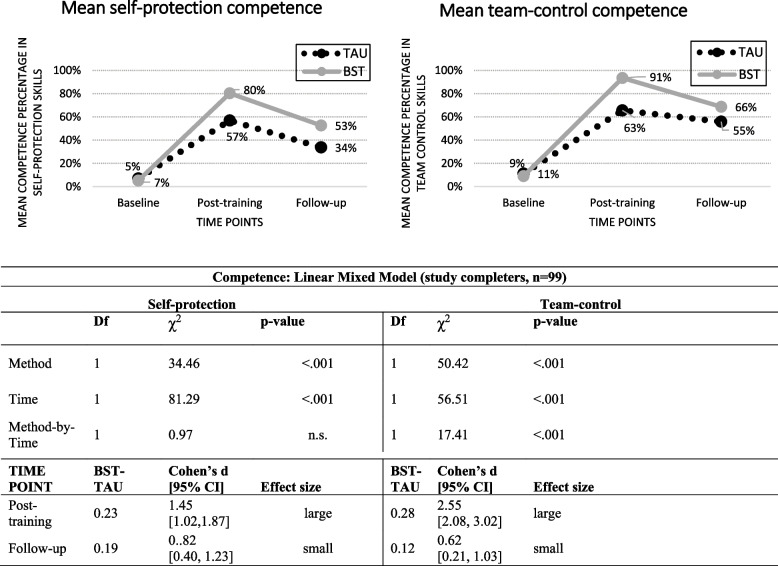
Observer-rated self-protection and team-control competence skills in TAU and BST across time-points

Figure 1 depicts the self-protection and team-control competence scores for the study completers (left and right sides, respectively). The hypothesis-testing results showed a significant difference by training Method (self-protection: *χ*^2^(1) = 34.46, *p* < 0.001; team-control: *χ*^2^(1) = 50.42, *p* < 0.001). There was also a significant decline between post-training and follow-up (Time) for both skill categories independent of Method (self-protection: *χ*^2^(1) = 81.29, *p* < 0.001; team-control: *χ*^2^(1) = 56.51, *p* < 0.001), and a significant Method-by-Time interaction independent of Method and Time for team-control skills (*χ*^2^(1) = 17.41, *p* < 0.001). BST-TAU comparisons showed no difference at baseline for either type of skill (not shown). However, BST was significantly better than TAU at both post-training (self-protection: Cohen’s *d* = 1.45 [1.02, 1.87], large effect size; team-control: Cohen’s *d* = 2.55 [2.08, 3.02]; large effect size) and follow-up (respectively – Cohen’s *d* = 0.82 [0.40, 1.23]; Cohen’s *d* = 0.62 [0.21, 1.03], both small effect sizes). For both methods, competence scores dropped between post-training and follow-up although not to the original baseline levels.

**Fig. 2:**
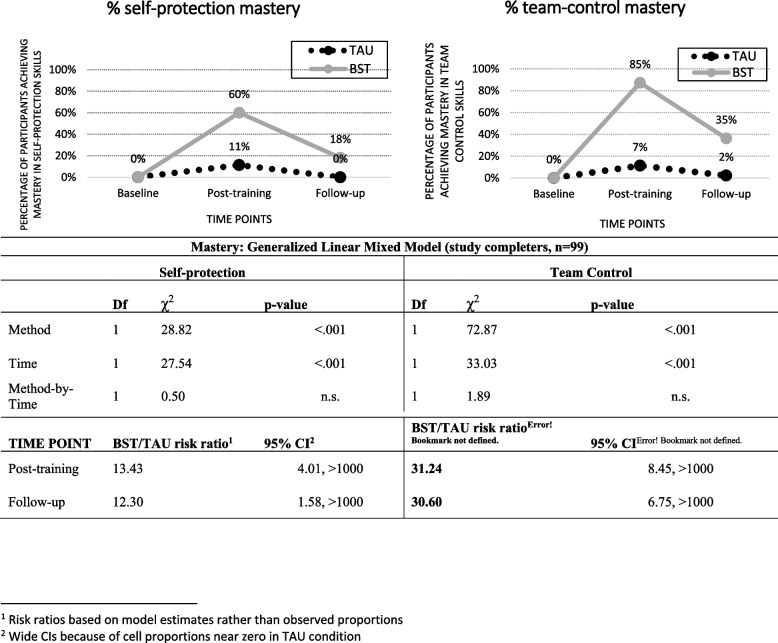
Observer-rated self-protection and team-control mastery (Predefined as 80% or better competence) by TAU and BST across time-points

The skill mastery results for the study completers are shown in Fig. 2. The mastery patterns paralleled the competence patterns in that BST was significantly better than TAU (self protection: *χ*^2^(1) = 28.82, *p* < 0.001; team-control: *χ*^2^(1) = 72.87, *p* < 0.001). There was also a significant Time effect independent of Method (self-protection: *χ*^2^(1) = 27.54, *p* < 0.001; team-control: *χ*^2^(1) = 33.03, *p* < 0.001). There were no significant interactions for either type of skill once the effects of Method and Time were accounted for. BST-TAU comparisons showed no difference in percent achieving Mastery at baseline (not shown) but large risk ratios at both post-training (self-protection: 13.43 [4.01, > 1000]; team-control: 31.24 [8.45, > 1000] and follow-up [self-protection: 12.30 [1.58, > 1000]; team-control: 30.60 [6.75. > 1000]).

**Fig. 3:**
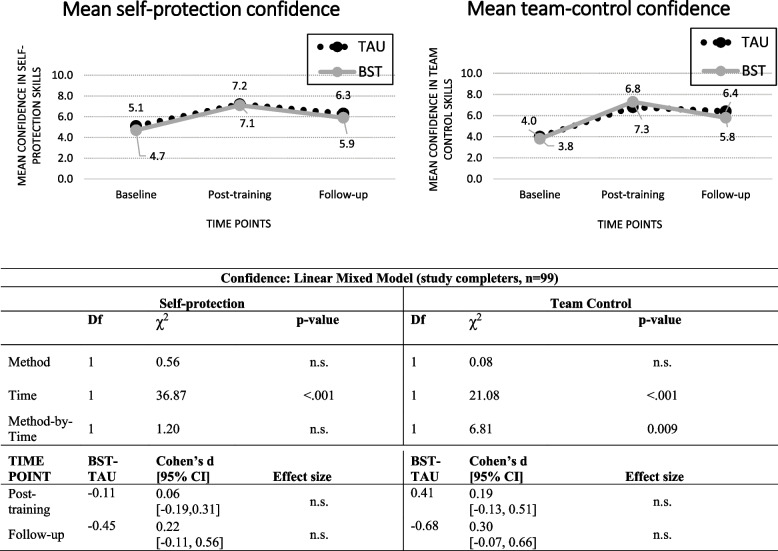
Self-rated self-protection and team-control confidence in TAU and BST across time-points

Confidence scores for the study completers are shown in Fig. 3. The only significant main effect was for Time (self-protection: *χ*^2^(1) = 36.87, *p* < 0.001; team-control: *χ*^2^(1) = 21.08, *p* < 0.001). For both skill categories, the scores increased between baseline and post-training and then dropped at follow-up but not to the original baseline levels.

To assess what impact the high no-show rate for the one-month follow-up could have had, we compared the completers and the non-completers on the six post-training outcomes (competence, mastery, and confidence for self-protection and for team-control). Non-completers had slightly lower scores than completers except for the two confidence measures where their self-assessments were higher (not shown). However, the only significant difference between the two groups was for self-protection competence means (0.70 vs 0.63, completers vs non- completers, *t*(195) = 2.40, *p* = 0.017).

**Fig. 4:**
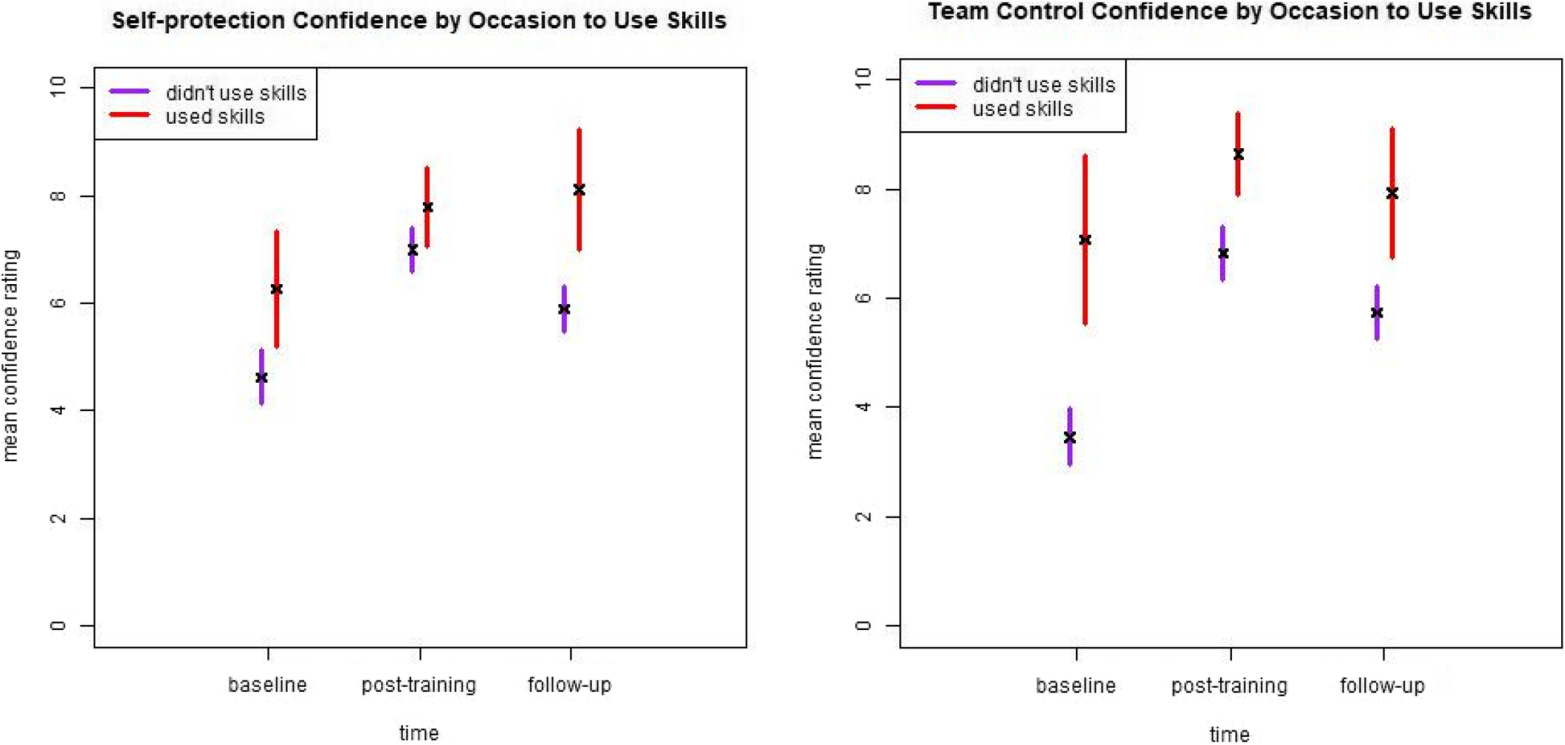
Self-rated self-protection and team-control confidence by occasion to use skills in the past month across time-points

In terms of past-month experience, few study completers reported events requiring self-protection (19 at baseline, 9 at follow-up) or team-control skills (14 at baseline, 14 at follow-up). Consequently, we only examined the presence or absence of experience without breaking it down by training method. We found non-significant results for both competence and mastery (not shown) but a potential impact on confidence for self-protection skills at follow-up and for team-control skills at baseline and post-training (Fig. 4).

4. Summary and discussion.

Our strongest finding was that BST was significantly better than TAU in improving the observed performance of self-protection and team-control skills. While follow-up scores decreased for both methods, BST scores remained higher than TAU scores. The impact of training on staff confidence differs from these patterns in that confidence scores improved noticeably at post-training and remained relatively high at follow-up. Further, our post-hoc analyses suggested that recent experience using safety skills might have a greater impact on confidence than on observed skill performance. We also found that training, regardless of method, was independently associated with improved observer-scored skills and self-reported confidence.

The better performance of BST is consistent with the fact that it incorporates training elements that are supported both by current educational and learning theories and evidence of effectiveness [46, 53–55]. While both BST and TAU can be considered ‘outcomes based’ [54], the key difference is the BST’s use of the checklist. Based directly on the desired behavioral outcomes, this tool simultaneously creates a common understanding because it is shared with the trainees, ensures consistent and systematic training across all BST trainees, pinpoints where immediate and personalized feedback is needed to either correct or reinforce performance, and tracks the number of correct repetitions required to meet mastery criteria as well as support retention [46, 56, 57]. By contrast, TAU does not use a checklist and the kind and amount of feedback or practice repetitions is left to the trainer’s discretion.

However, there are at least two questions regarding whether BST produced the expected results. The BST framework requires continued rehearsal and feedback until a specified performance criterion is reached [34]. However, our mandatory safety training had practical, unmodifiable constraints. The institution required the safety-training sessions be completed in 3.5 h which meant that BST trainers were limited in their ability to use the more stringent performance criteria described in the literature. For example, it was not practical to set the performance criterion at higher than 80 percent. In addition, all BST completers were able to demonstrate 80-percent correct performance for each skill at least once, but not all were able to demonstrate five consecutive, correct executions within the allotted time. If the requirement of five in a row at 80% or higher had been implemented, then the post-training scores (and potentially the 1-month follow-up scores) for the BST completers could have been higher.

A second question is what level of skill retention should be expected at follow-up. The BST scores at one-month follow-up constituted 66% and 73% of the competence scores at post-training (self-protection and team-control, respectively) and 30% and 41% of the mastery percentages at post-training (self-protection and team-control, respectively). Although BST and elements of performance feedback models have been found to be effective in staff training with successful retention over time [58–62], finding appropriate comparators for our study was challenging because there are no studies where BST has been used for training such a large and diverse group of staff. Further, as noted above, the body of workplace violence prevention literature has not consistently focussed on retention. However, the broader training and education literature does suggest that our results are consistent with or somewhat lower than those from other studies. Offiah et al. [63] found that 45 percent of medical students retained the full set of clinical skills 18 months after completing simulation training, and Bruno and colleagues [64] found published retention rates ranging between 75 and 85 percent across time periods between four to 24 months and across diverse disciplinary fields. Regardless of the comparators, the loss in skill performance after one-month post-training is a concern.

Our interpretation is that reliance on a single session, even with highly structured and competency-based methods, is not adequate particularly in the context of managing distressing events. Efforts should be made to allow for flexibility with respect to setting higher thresholds for success despite organizational restraints for staff training. Furthermore, settings that require these skills to be performed more reliably for both patient and staff safety (e.g., emergency departments, acute care settings, security services) should consider on-the-job feedback or booster sessions based on objective assessments of skill rather than on pre-set amounts of time (e.g. annual refresher). This would be more consistent with the BST literature, as on-the-job training should occur based on an evidence-based approach.

Our finding of a differential impact of training on confidence versus demonstrable skills is consistent with a long-standing, substantial body of research examining the relationship between self-assessment and objective measures of learning [28, 65, 66]. The pattern of non-existent, weak, or even inverse relationships between the two has been shown for a variety of medical staff trainee and education learner groups [28, 29, 67–72]. Consequently, many researchers recommend either not using self-assessments at all or at least ensuring that objective measures are also collected (e.g.,[64, 65]).

The literature does offer some hypotheses for why this discrepancy occurs and, further, why self-assessment continues to be used in medical education and training despite the robust evidence that it does not accurately reflect learning. Katowa-Mukwato and Banda [70] in a study of Zambian medical students suggest that fear of revealing their weaknesses led to a negative correlation between self- and objective-ratings. Persky, et al. [69] reference the theory of ‘metacognition’ – defined as ‘thinking about thinking’ (p. 993, [69] – and the ‘Dunning-Kruger’ effect that the ability to recognize competence (i.e., accurate metacognition) is unevenly distributed. There is also discussion as to why these measures continue to be used and suggestions of how best to use them. Yates et al. [65] suggest that ease of collecting this information is a factor. More complex and nuanced explanations are offered by Lu, et al. [66] and Tavares, et al. [73] who note that self-assessment is an important component in theories of learning and evaluation and that self-perception and self-reflection (particularly when objective findings are shared) are critical ingredients for supporting medical and continuing profession education in a self-regulating profession.

Because the goal of our study was to assess the effectiveness of two training methods, we did not collect information or have the opportunity to explore any of these potential reasons for why self-reported and objective measures are discrepant or to evaluate the best use of that discrepancy. The modest contributions that our study adds are that selecting a higher-risk setting, including non-nursing healthcare professionals, using a more rigorous study design (as recommended by Geoffrion, et al. [29]), and attempting to account for recent experience do not appear to alter this pattern.

The major strength of our study is its design. Currently, we have identified only one other study evaluating the impact of BST training for clinical staff using a randomized control trial design [41]. Other strengths are our inclusion of a large percentage of non-nursing, direct-care staff, our use of both self-reported and observer-assessed outcome measures, and our findings regarding retention. These strengths allow us to add to the evidence base already established in the literature.

However, interpretation of our results should consider several limitations. Conducting a research study on full-time clinical staff during a pandemic meant that a high percentage of those consenting to be in the study did not complete their 1-month follow-up assessment. The reported reasons for missing the third assessment (unit restrictions or short staffing because of the pandemic) are consistent with the demographic differences between completers and non-completers in that they were more likely to be nurses or working on inpatient units. Our comparison of the post-training scores of the completers and non-completers suggested that the no-shows had slightly lower post-training observed skill performance (but slightly better confidence ratings). If we had managed to assess the non-completers at follow-up, our reported findings may have been diluted although it is unlikely that this would have completely negated the large effect sizes.

The time constraints on the mandatory training meant that we were unable to fully apply either the BST mastery criteria commonly reported in the literature (i.e., three correct, consecutive executions [28, 47] or the one we would have preferred (i.e., five correct executions). While this type of limitation is consistent with the pragmatic nature of our design, it likely had an impact on our findings in terms of potentially lowering the post-training BST competency and mastery scores and, perhaps more importantly, contributing to the lower retention rates at 1-month follow-up [56].

The 45-percent refusal rate by the training registrants is another concerning issue. Anecdotal reports from the training team were that the response rate was very low at the start of the study because many of the new hires were nervous about being videotaped (a specific comment reported was that it reminded some of the new graduates of ‘nursing school.’) and were unsure of the purpose of the study. The team then changed to a more informal, conversational introduction describing the need for the study as well as reassuring attendees that it was the training, not the participants, that was being evaluated. The team’s impression was that this improved the participation rate. The participants and non-participants were not statistically different in terms of their expected patient contact and department role. However, we cannot preclude that there may have been systematic biases for other unmeasured characteristics.

Another limitation, as identified by Price, et al. [28], is that we used artificial training scenarios, though this may be unavoidable given the low frequency of aggressive events and the ethics of deliberately exposing staff to these events. Also, we only measured the skills directly related to handling client/patient events. We were not able to access information on event frequency or severity, staff distress and complaints, or institutional-level measures such as lost workdays due to sick leave, staff turnover, or expenditures [29, 33]. A further gap, which is important but difficult to assess, is whether there is any impact of staff safety training on the clients or patients who are involved.

Given these strengths and limitations, we see our study as adding one piece of evidence that needs to be a) confirmed or disconfirmed by other researchers in both the same and different settings and b) understood as part of a complex mix of ingredients. Specific areas for further research arising directly out of our findings include evaluating whether less constrained training time would improve attainment of skill mastery, exploration and evaluation of methods to increase skill retention over time, and, most importantly but also more difficult to assess, the impact on patients and clients of staff safety skills training. More evidence on these fronts will hopefully contribute to maintaining and improving workplace safety.

### Supplementary Information

**Additional file 1.**

**Additional file 2.**

## Data Availability

The dataset generated and analysed during the current study is not publicly available due to the fact that it is part of a larger internal administrative data collection but is available from the corresponding author on reasonable request.
