# Supplementary material for: Behavioral skills training for teaching safety skills to mental health service providers compared to training-as-usual: a pragmatic randomized control trial
Source: BMC Health Serv Res. 2024 May 17;24:639. doi: 10.1186/s12913-024-10994-1 (PMC11102142; doi:10.1186/s12913-024-10994-1)
Supplement: Supplementary file 1 — Additional file 1. [file 12913_2024_10994_MOESM1_ESM.docx]

APPENDIX A: Self-protection and team-control skills

**Safety Skills Curriculum (for both TAU and BST training)**

**Self-Protection Skills**-

1- and 2-Hand Attempt to Push/ Punch/Grab Defense,

1- or 2-Hand Cross-Arm Grab Defense,

Roundhouse or Open-Handed Slap Defense,

Same-Side Grab Defense,

2-Handed Front Choke Defense,

Rear Choke Defense,

**2- 5 Person Team Control (Physical Restraint) Skills**-

Level 1, Two Person Team Control

Level 2, Two Person Team Control

Level 3, Two Person Team Control

Additional Hand Controls

Anchor

APPENDIX B: Example of BST checklist

| **Two Handed Front Choke Defense** | |  |  |  |
| --- | --- | --- | --- | --- |
| **Observed Skills** | | Baseline | Post Training | Follow-up |
| 1 | Turtle your neck |  |  |  |
|  | a. Bring chin down to chest | ✔ X N/A | ✔ X N/A | ✔ X N/A |
|  | b. Shrug your shoulder up towards your ears | ✔ X N/A | ✔ X N/A | ✔ X N/A |
| **If defender is stepping out or back to right side** | | | | |
| 2 | Step back or to the right with right leg | ✔ X N/A | ✔ X N/A | ✔ X N/A |
| 3 | Bring left hand up in front of face | ✔ X N/A | ✔ X N/A | ✔ X N/A |
| 5 | Bring right hand up on outside of aggressor left hand | ✔ X N/A | ✔ X N/A | ✔ X N/A |
| 6 | Left hand secure aggressor's left hand (on your neck/shoulder) | ✔ X N/A | ✔ X N/A | ✔ X N/A |
| 7 | Right hand comes up and makes full circle/windmill to break the grab | ✔ X N/A | ✔ X N/A | ✔ X N/A |
| 8 | Left leg rotate/step back | ✔ X N/A | ✔ X N/A | ✔ X N/A |
| 9 | Right hand on the aggressor's left shoulder | ✔ X N/A | ✔ X N/A | ✔ X N/A |
| 10 | End up at a 45 degree angle from aggressor | ✔ X N/A | ✔ X N/A | ✔ X N/A |
| 11 | After ending up in 45 degree angle, disengage/let go within 1 second | ✔ X N/A | ✔ X N/A | ✔ X N/A |
| **Total correct** | |  |  |  |
| **% correct** | |  |  |  |

✔= observed X=not observed N/A=not applicable

APPENDIX C: Procedure for BST training

*Training Procedure*

Two Education Specialists are present during the training. The training is guided by the behavioural skills training model which includes the following five stages:

1. **Instruction**: Attendees are provided with a written step-by-step instruction of each skill

2. **Modeling:** both the instructors demonstrated the skills, and a video model demonstrating the skills was provided

3. **Rehearsal:** Participants are provided with an opportunity to rehearse in front of TIDES Education Specialist

i. If attendee is observed to be having difficulties with the verbal instruction from the instructor, staff is prompted to review a video of skill.

4. **Feedback** Facilitators provide feedback to the attendee about each of the steps listed in the **competence** checklists of physical skills:

i. Positive Verbal Feedback regarding correct response

- Facilitator provides positive behaviour specific verbal praise for correct steps completed (i.e. “Good job stepping to the right!” )

- Facilitator provides gesture i.e. both or one thumb up

ii. Corrective Verbal feedback regarding missed or incorrect steps

- Facilitator provides corrective feedback statement outlining missed step (e.g. “great job stepping to the left, next time also bring your hands up.”)

- Facilitator provides opportunity for participant to return to rehearsal (i.e. step-by-step instruction is provided with video model if required)

**5.** **Competence Checklist** (for in-training purposes): Facilitator asks attendee to demonstrate skill with no instruction

i. Staff are expected to demonstrate each skill at 80-100% competence for five consecutive times

APPENDIX D: Self-rated Confidence Questions

1. If a patient started to behave aggressively towards you, how confident would you be that you could protect yourself? (please use a check mark √ to identify your response):

| **Not at all confident**  **0** | 1 | 2 | 3 | 4 | 5 | 6 | 7 | 8 | 9 | **Extremely confident**  **10** |
| --- | --- | --- | --- | --- | --- | --- | --- | --- | --- | --- |
|  |  |  |  |  |  |  |  |  |  |  |

|  |  |  |  |  |
| --- | --- | --- | --- | --- |

1. If you needed to physically transfer or restrain an agitated patient, how confident would you be that you could do so? (please use a check mark √ to identify your response):

| **Not at all confident**  **0** | 1 | 2 | 3 | 4 | 5 | 6 | 7 | 8 | 9 | **Extremely confident**  **10** |
| --- | --- | --- | --- | --- | --- | --- | --- | --- | --- | --- |
|  |  |  |  |  |  |  |  |  |  |  |

|  |  |  |  |  |
| --- | --- | --- | --- | --- |
